# Supplementary material for: Selected Psychosocial Factors, Nutritional Behavior, and the Analysis of Concentrations of Selected Vitamins in Patients with Cardiovascular Diseases
Source: Nutrients. 2024 Jun 14;16(12):1866. doi: 10.3390/nu16121866 (PMC11206887; doi:10.3390/nu16121866)
Supplement: Supplementary file 1 [file nutrients-16-01866-s001.zip › nutrients-3046360-supplementary.pdf]

Table S1. Prevalence of cardiovascular diseases in each cluster after applying data reduction.

Parameter analyzed	Disease occurrence	Cluster 1 (n=26)		Cluster 2 (n=35)		*p*-value[a]
		N	%N	N	%N	
**Atherosclerosis**	yes	0	0	8	13.1	**0.016**
	no	26	42.6	27	44.3	
**Arterial hypertension**	yes	26	42.6	19	31.2	**<0.001**
	no	0	0.00	16	26.2	
**Ischemic heart disease**	yes	26	42.6	19	31.2	**<0.001**
	no	0	0.00	16	26.2	
**Infarcts of various organs**	yes	0	0.00	15	24.6	**<0.001**
	no	26	42.6	20	32.8	

Legend: [a] - Fisher's exact test (expected values different from 0); Cluster 1 - patients with hypertension, ischemic heart disease without atherosclerosis and infarcts of various organs; Cluster 2 - included indicated co-occurrence of atherosclerosis or infarcts of various organs with any of the other disease; statistically significant differences for individual clusters are in bold.

Table S2. Analysis of selected lifestyle factors-use of stimulants and physical activity in the study group of patients.

Parameter analyzed		The use of	Cluster 1		Cluster 2		*p*-value[a]
			N	%N	N	%N	
**Use of stimulants**	**Smoking**	yes, I am an addict	9	14.8	19	31.2	**<0.001**
		yes, occasionally	1	1.64	4	6.56	
		smoked in the past	10	16.4	16	26.2	
		don't smoke	2	3.28	0	0	
	**Alcohol consumption**	yes, I am an addict	1	1.64	1	1.64	>0.05
		yes, occasionally	16	26.2	26	42.6	
		drank in the past	5	8.2	10	16.4	
		don't drink	0	0	2	3.28	
	**Other stimulants**	yes	0	0	0	0	>0.05
		no	21[#]	34.4	38[#]	62.3	
	**Physical activity**	high	1	1.64	5	8.2	>0.05
		medium	10	16.4	19	31.2	
		low	11	18	15	24.6	

Legend: N – number of patients; [a] Pearson's χ^2 test; Cluster 1 - as-signed non-smokers and past smokers,; Cluster 2 - occasional smokers and addicts; bold values denote statistical significance at the p<0.05 level; [#] - o response given.

Table S3. Analysis of current health and psychophysical functioning in each cluster after applying data reduction.

Parameter analyzed	Restrictions	Cluster 1		Cluster 2		*p*-value[a]
		N	%N	N	%N	
**Fatigue too fast**	yes	5	8.20	43	70.5	**<0.001**
	no	12	19.7	1	1.64	

Parameter analyzed	Restrictions	Cluster 1		Cluster 2		*p*-value[a]
		N	%N	N	%N	
**Need to reduce working hours**	yes	0	0.00	42	68.9	**<0.001**
	no	17	27.9	2	3.28	
**Worse mood**	yes	8	13.1	38	62.3	**0.003**
	no	9	14.8	6	9.84	
**Difficulties in performing daily activities**	yes	0	0.00	35	57.4	**<0.001**
	no	17	27.9	9	14.8	

Legend: [a] - Fisher's exact test (expected values different from 0); Cluster 1 - no impact of health status on functioning; Cluster 2 limitations in acting, fast fatigue, worse mood, problems with performing daily activities, need to reduce working time; statistically significant differences for individual clusters are bolded.

Table S4. Analysis of emotional states in the study group.

Parameter analyzed	Perception	Cluster 1		Cluster 2		*p*-value[a]
		N	%N	N	%N	
**Nervousness**	never	13	21	2	3	**<0.001**
	very rarely	5	8	11	18	
	from time to time	1	2	11	18	
	often	0	0	9	15	
	very often	0	0	8	13	
**Ability to be comforted**	never	19	31	13	21	**<0.001**
	very occasionally	0	0	17	28	
	from time to time	0	0	3	5	
	often	0	0	5	8	
	very often	0	0	3	5	
**Increased energy/willingness to live**	never	19	31	26	43	>0.05
	very occasionally	0	0	10	16	
	from time to time	0	0	2	3	
	often	0	0	2	3	
	very often	0	0	1	2	
**Calming/reassurance**	never	19	31	10	16	**<0.001**
	very occasionally	0	0	16	26	
	from time to time	0	0	9	15	
	often	0	0	6	10	
	very often	0	0	0	0	
**Breaking down/sadness**	never	16	26	7	12	**<0.001**
	very occasionally	3	5	12	20	
	from time to time	0	0	13	21	
	often	0	0	5	8	
	very often	0	0	3	5	
**Increased sense of happiness**	never	19	31	23	38	**0.018**
	very occasionally	0	0	11	18	
	from time to time	0	0	4	7	
	often	0	0	2	3	
	very often	0	0	1	2	
**More frequent fatigue**	never	2	3	0	0	**<0.001**

very occasionally	4	7	2	3
from time to time	9	15	8	13
often	4	7	13	21
very often	0	0	18	30

Legend: N – number of patients; [a] Pearson's χ^2 test; Cluster 1 - reduced capacity for comfort and less energy/willingness to live, not indicating calmness and feelings of happiness, not showing illness-related breakdown; Cluster 2 - patients who showed limtations in functioning; bold values denote statistical significance at the $p<0.05$ level;
